# Study of Effects of Topical Fluorometholone on Tear MCP-1 in Eyes Undergoing Trabeculectomy: Effect on Early Trabeculectomy Outcomes in Asian Glaucoma Patients

**DOI:** 10.3390/jcm14228057

**Published:** 2025-11-13

**Authors:** Olivia Shimin Huang, Jackie Jia Lin Sim, Hla Myint Htoon, Annabel Chee Yen Chew, Rachel Shujuan Chong, Rahat Husain, Shamira Perera, Tina T. Wong

**Affiliations:** 1 Singapore National Eye Centre, Singapore 168751, Singapore; 2 Singapore Eye Research Institute, Singapore 169856, Singapore; 3 Department of Ophthalmology, Yong Loo Lin School of Medicine, National University of Singapore, Singapore 117597, Singapore; 4 Ophthalmology and Visual Sciences Academic Clinical Program, Duke-NUS Medical School Singapore, Singapore 169857, Singapore

**Keywords:** glaucoma, trabeculectomy, wound modulation, steroids

## Abstract

**Objectives:** We aimed to determine if a 2-week pre-operative course of fluorometholone (FML) eyedrops in chronically medicated glaucoma patients reduces the levels of the pro-inflammatory cytokine Monocyte Chemoattractant Protein 1 (MCP-1) and improves early post-operative outcomes after trabeculectomy or phaco-trabeculectomy. **Methods:** We conducted a single-center, unmasked, prospective pilot interventional case series of 36 patients with glaucoma who received a 2-week course of FML eyedrops prior to undergoing trabeculectomy. A multiplex bead assay was used to quantify the presence of MCP-1 levels in tear samples before and after the use of FML eyedrops, and 307 eyes without treatment with topical FML served as historical controls. Clinical outcome measures of early post-operative outcomes included IOP and additional post-operative interventions (i.e., needling, glaucoma medications, and surgery) required to achieve the desired IOP at 6 months. **Results:** Out of 36 patients who received FML, 19 patients had a low MCP-1 level (<250 pg/mL/mg) at baseline, which did not significantly change after using FML, and were excluded from analysis. Of the 17 remaining patients, propensity score-matched analysis was conducted to compare them with 17 patients who did not receive FML, matching for the variables of age, gender, ethnicity, diagnosis, longest glaucoma medication duration, and surgery type. Patients with FML treatment had lower odds of requiring any post-operative intervention (including needling, surgery, or IOP-lowering medications) (OR 0.22, CI 0.049–0.95, *p* = 0.042) compared to patients who did not have pre-operative FML treatment. **Conclusions:** In patients with higher levels of MCP-1 pre-operatively, the use of FML for 2 weeks pre-operatively improved their early post-operative outcomes following trabeculectomy or phaco-trabeculectomy.

## 1. Introduction

Trabeculectomy is a form of glaucoma filtration surgery that lowers intraocular pressure (IOP) by creating a drainage pathway from the anterior chamber to the subconjunctival space. It is used for treating medically uncontrolled glaucoma where a low target IOP is required, with a favorable cost–benefit analysis compared to other surgical techniques [1]. However, post-operative conjunctival scarring can result in bleb failure [1], rates of which can be as high as 35–43% [2,3]. A major risk factor for this is the use of chronic topical IOP-lowering medications with their accompanying preservatives [4] prior to trabeculectomy, which induces higher numbers of inflammatory cells and fibroblasts in the conjunctiva [5,6], resulting in conjunctival fibrosis [7]. The pro-inflammatory state of the conjunctiva can be detected through the analysis of cytokines that are secreted into tears [8]. Amongst these cytokines, Chong et al. previously reported that elevated MCP-1 levels resulted in a greater propensity towards subconjunctival scarring in the early post-operative period of 6 months [8].

Wound modulation is crucial in preventing the excessive scarring that leads to bleb failure [9,10,11] after trabeculectomy. Topical corticosteroids can be used peri-operatively [12,13] to improve bleb survival [14,15,16], and their beneficial effects have been shown to persist up to 10 years post-trabeculectomy [17]. Steroids reduce the inflammatory response by mitigating the activity of leucocytes, inhibiting phagocytosis, decreasing the response to presented antigens, and reducing downstream production of inflammatory mediators. They also reduce vascular permeability to minimize the release of growth factors, the production of clot and fibrin, and cellular migration to the site of injury, which may result in the inhibition of fibroblastic activity [14,18].

While steroids are routinely [19,20] used in the post-operative course [18], opinions vary regarding the use of steroids before trabeculectomy. A survey of glaucoma specialists reported 85.9% agreed that it was “beneficial”, and only 48.4% felt it was “necessary” [21]. Previous studies have provided evidence for the beneficial effect of pre-operative steroids but differed in the duration and type of topical steroids used pre-operatively [22,23,24]. A randomized controlled trial (RCT) with 54 patients from Belgium [12] comparing the use of topical fluorometholone (FML), ketorolac, and placebo prior to trabeculectomy found that topical FML used for 1 month before surgery was associated with a reduced likelihood of post-operative needling (5% vs. 6–41%) and a significantly lower proportion of patients required post-operative glaucoma medications to reach the target IOP at 1 year (0% vs. 18–24%). Another RCT from Russia randomized 80 patients to instill either nepafenac, dexamethasone, or a combination of both for 2 weeks before surgery and found that pre-operative local anti-inflammatory therapy helped to increase the 1-year complete success rate after trabeculectomy (65% for nepafenac, 75% for dexamethasone, 80% for combination), compared with controls (50%) [13]. These studies were conducted in Caucasian populations, whose propensity toward scarring differs from other racial populations. There have not been any previous studies in an Asian population to determine whether the pre-operative use of topical steroids might be beneficial in improving early surgical outcomes, nor are there any reports on the effect of pre-operative steroids on the pro-inflammatory state of the conjunctiva in glaucoma patients.

The aim of our study was to investigate the effect of a 2-week course of FML on pre-operative MCP-1 levels in the tears of medicated Asian glaucoma patients scheduled for trabeculectomy or phaco-trabeculectomy and to determine their early surgical outcomes post-operatively.

## 2. Materials and Methods

This study was a single-center, unmasked, prospective interventional pilot case series, which included glaucoma patients on IOP-lowering topical medication and scheduled for trabeculectomy or phaco-trabeculectomy at the Singapore National Eye Centre from 2019 to 2020. We excluded patients with any of the following criteria at baseline: previous major ocular surgery (not inclusive of phacoemulsification), previous documented steroid response, allergy to steroids or the preservative benzalkonium chloride, a history of herpetic keratitis, patients with only one functional eye, pregnant or lactating patients, patients with secondary glaucoma, patients receiving oral steroids, patients receiving pre-existing topical steroids in the eye scheduled for trabeculectomy, patients exclusively using preservative-free eyedrops pre-operatively, and those aged below 21 years old or not able to give informed consent. Informed written consent pertaining to the donation and storage of human fluid samples was obtained from all subjects, and the study was conducted in accordance with the Declaration of Helsinki and after approval from the local Centralised Institutional Review Board at SingHealth (CIRB Ref: 2018/2626).

The glaucoma subtypes, namely, primary open-angle glaucoma, normal tension glaucoma, and primary angle closure glaucoma, were diagnosed by clinicians according to the European Glaucoma Society guidelines [25]. Trabeculectomy was indicated in the presence of poorly controlled glaucoma where there was suboptimal IOP control despite maximal medical therapy, progressive visual field loss, or both.

### 2.1. Fluorometholone Pre-Operative Regime

Patients were given FML 0.1% eyedrops to be taken four times a day for 2 weeks immediately prior to their surgery.

### 2.2. Tear Collection and Extraction of Tear Protein from Schirmer’s Strips

Tears were collected from patients before surgery at 2 time points before the patient’s scheduled surgery, once just before the use of the FML and once after the use of FML for 2 weeks (just prior to commencing the surgery). A Schirmer’s tear test strip was inserted into the lower fornix of the unanesthetized eye once to collect tears. After 5 min, the strip was removed from the eye and the length of the wetted area of the strip was measured. Study subjects with wetted areas of less than 5 mm in 5 min were excluded from this study. The Schirmer’s strips were frozen immediately at −80° C after collection and maintained until analysis.

During extraction, the wetted area of each strip was cut into small pieces, which were soaked in enough phosphate-buffered saline to cover them completely for 3 h to elute tear proteins. Total tear protein was then measured using a BCA protein assay (Pierce Biotechnology, Inc., Rockford, IL, USA).

### 2.3. Analysis of MCP-1 Cytokine in Human Tears with the Bio-Plex System

Color-coded 5.6-μm polystyrene beads coupled covalently with specifically directed antibodies (Bio-Plex Human Cytokine; Bio-Rad Laboratories, Hercules, CA, USA) were allowed to react with 50 μL of each sample containing an unknown amount of cytokine, or with a standard solution containing a known amount of cytokine, at room temperature for 1 h.

After performing a series of washes to remove unbound protein, a biotinylated detection antibody specific to a different epitope on the cytokine was added to the beads and was incubated at room temperature for 30 min. Streptavidin-phycoerythrin, which binds to the biotinylated detection antibodies, was used to detect the reaction mixture. The flow-based Bio-Plex suspension array system then identified and quantified each specific reaction based on bead color and fluorescence, using fluorescently labeled reporter molecules associated with each target protein. Unknown cytokine concentrations were calculated automatically by Bio-Plex Manager software (Version 6.1.0.727) using a standard curve derived from a recombinant cytokine standard. MCP-1 cytokine levels were corrected for initial total protein concentration of each tear sample during analysis and were expressed in picograms per milliliter per milligram (pg/mL/mg).

### 2.4. Outcome Measures

The primary outcome measure in our study was the level of tear MCP-1 in patients before and after the use of FML eyedrops. Secondary outcome measures included the post-operative outcomes, which included the final IOP and rates of post-operative interventions at 6 months (inclusive of bleb needling with subconjunctival injection of anti-fibrotic agents, addition of IOP-lowering medications, or further surgical interventions to achieve the desired IOP based on clinician discretion). We compared these outcomes with retrospective data from our center for patients who underwent trabeculectomy or phaco-trabeculectomy but did not receive pre-operative FML eyedrops during the same time period.

### 2.5. Power Calculations

Sample size calculation was conducted with PASS Sample Size software (2022 Version 22.0.8). The primary outcome variable used for the power calculation was the percentage of patients requiring IOP-lowering medication to reach the target IOP based on an RCT by Breusegum et al. [12] comparing the use of pre-operative topical FML vs. controls in patients undergoing trabeculectomy. The assumed effect size was based on their results; the percentage of patients requiring IOP-lowering medication to reach the target IOP at one year was 24% for the control group vs. 0% for the steroid group. Although the reference stated 0%, we assumed that 0% would be too optimistic, and hence we used a value of 3% for our calculations. Group sample sizes of 34 in each group achieved an 80% power to detect a difference between the group proportions of −0.2100. The proportion in the treatment group was assumed to be 0.2400 under the null hypothesis and 0.0300 under the alternative hypothesis. The proportion in the control group was 0.2400. The test statistic used was the two-sided Z test with pooled variance. The significance level of the test was set as 0.0500. The significance level actually achieved by this design was 0.0541.

### 2.6. Statistical Analysis

We compared the group receiving FML (*n* = 36) with the group that did not receive FML (*n* = 307). An independent T test was conducted for continuous variables and Pearson’s Chi-squared test/Fisher’s exact tests were conducted for categorical variables. Observations from a scatterplot from individual patients revealed that patients with a low baseline MCP-1 level <250 did not result in a significant change in MCP-1 levels after using FML. Therefore, a subgroup with pre-operative MCP-1 levels <250 was excluded from the FML group in further sub-analysis, leaving 17 patients for analysis. In order to correct for significant baseline pre-operative differences between the FML and non-FML group, which could confound our results, propensity score matching (PSM) 1:1 for this subgroup (*n* = 17) was performed with the non-FML group for the variables of age, gender, ethnicity, diagnosis, longest glaucoma medication duration, and surgery type, with psmatch2 propensity matching with STATA ver.16 software (Total *n* = 34). Due to the number of variables, caliper adjustment was not suitable. A non-caliper matching without replacement was instead conducted. The propensity score variance ratio of the 2 groups ranged from 0.64 to 1.17 and was within the limits of 0.36 and 2.70, with a mean bias of 19.2 (*p* = 0.850). Univariate logistic regressions were conducted for the matched data. A 2-sided alpha level of 0.05 was considered statistically significant. IBM SPSS statistics v.29 was used for the analyses.

## 3. Results

Out of an initially recruited 49 patients who were given topical FML pre-operatively, 13 patients withdrew from the study, for reasons including cancelation of the trabeculectomy on the intended operation day for uncontrolled hypertension, bradycardia, or complications during cataract surgery (*n* = 7), disruptions due to COVID-19 resulting in the ceasing of research-related activities (*n* = 3), refusal to return for follow-up (*n* = 2), and the patients not using the FML eyedrops pre-operatively (*n* = 2).

Table 1 shows a comparison of the remaining 36 patients in our study who received FML eyedrops with retrospective data from our center of 307 patients who did not receive FML eyedrops. There were no significant differences between the groups, respectively, in terms of age (65.6 ± 11.3 vs. 67.9 ± 12.1, *p* = 0.278), gender (52.8% vs. 65.8%, *p* = 0.143), or type of surgery; about 33.3% and 27.7% had a trabeculectomy while the remaining patients had a phaco-trabeculectomy (*p* = 0.477). There was also no difference in intra-operative complications (2.8% vs. 0.7%, *p* = 0.213) between the groups; there was one posterior capsule rupture in each of the groups and one retrobulbar hemorrhage in the group that did not receive FML. There were some racial differences in the Asian ethnic subtypes, where there were fewer Chinese and Eurasian patients and more Malay and Indian patients among those receiving FML compared to those who did not (*p* = 0.035). The FML group had more patients with NTG compared to those with POAG and PACG (55.6%, 22.2% and 19.4%, respectively), while the non-FML group had more patients with POAG compared to those with PACG and NTG (55.4%, 27.7%, 16.3%) at baseline. The types and numbers of pre-operative glaucoma medications were similar in both groups, but the FML group had a significantly lower mean duration of medications prior to surgery (32.9 ± 30.2 vs. 67.2 ± 57.8 months, *p* < 0.001). None of the patients in the FML group reported any side effects from FML.

**Figure 1:**
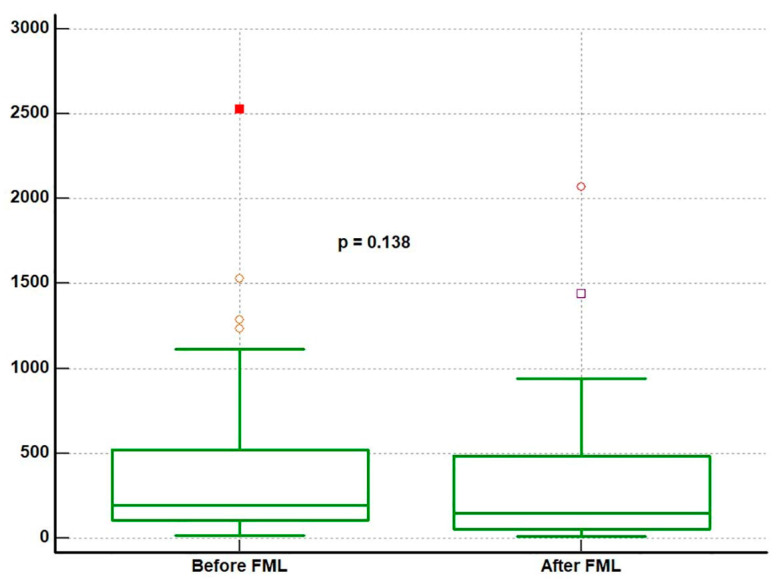
Box plot comparing the mean and 95% CIs of MCP-1 levels before and after using FML (*n* = 36) using a paired T-test. The dots in the box plot are outliers, and indicate the spread of the data. These patients had higher MCP-1 levels, possible reasons of which have been discussed in Section 4.

**Figure 2:**
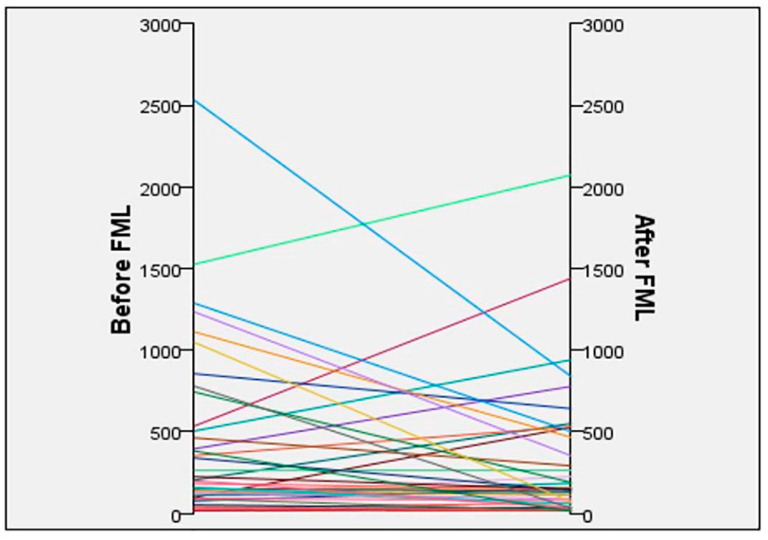
Scatterplot of MCP-1 levels detected in tears of individual subjects before and after use of FML (*n* = 36).

Figure 1 shows the box plot of the levels of MCP-1 before and after receiving FML. There was a general trend of a reduced MCP-1 level after receiving FML treatment; mean MCP-1 levels were 423.4 ± 523.9 pg/mL/mg at baseline, and 314.1 ± 442.4 pg/mL/mg after the use of FML for 2 weeks. A scatter plot showed that for those with lower MCP-1 levels (<250 pg/mL/mg) pre-operatively, MCP-1 levels remained at a similar level after using FML (Figure 2).

At 6 months post-trabeculectomy, initial analysis showed that the number of needlings was lower in patients who received FML compared to those without (1.2 ± 0.63 vs. 1.5 ± 0.74, *p* = 0.02) (Table 2A). Further sub-analysis (Table 2B) using propensity score matching was performed to analyze a subset of patients who had higher pro-inflammatory cytokines pre-operatively, by excluding patients with pre-operative MCP-1 levels <250 pg/mL/mg, based on exploratory post hoc observations of the scatterplot in Figure 2; those with MCP-1 levels <250 pg/mL/mg at baseline did not have much further reductions in their MCP-1 levels after FML treatment. After propensity score matching, the mean age of the FML group (*n* = 17) was 71.88 years, and that of the non FML group (*n* = 17) was 73.71 years (*p* = 0.449). The mean duration of glaucoma medications used pre-operatively was 31.29 months (FML group) and 34.79 months (non-FML group) (*p* = 0.763). Data could not be analyzed for the variable of “any further surgical intervention” due to collinearity. Patients with FML treatment had lower odds of requiring any post-operative intervention (including needling, surgery, or IOP-lowering medications) (OR 0.22, CI 0.049–0.95, *p* = 0.042) compared to patients who did not have pre-operative FML treatment.

We analyzed the differences between patients with higher pre-operative MCP-1 levels and those with lower levels (<250 pg/mL/mg) (Table 3) and found that those with higher MCP-1 levels were more likely to be older (71.88 ± 6.5 vs. 59.95 ± 11.8 years, *p* < 0.001). There were no significant differences between groups in terms of gender, race, duration of medications, number of different medications at the time of surgery, glaucoma diagnosis, or post-operative outcomes at 6 months.

## 4. Discussion

Our study demonstrated that in a subset of patients who have higher MCP-1 levels pre-operatively, the pre-operative use of FML eyedrops for two weeks improved outcomes following trabeculectomy and phaco-trabeculectomy, resulting in lesser odds of requiring any post-operative intervention (including needling, surgery, or IOP-lowering medications) (OR 0.22, CI 0.049–0.95, *p* = 0.042). This demonstrates the beneficial effects of pre-operative FML in selected Asian patients. Our study is also the first to report that a shorter duration of FML of 2 weeks may be sufficient to yield beneficial effects in the early post-operative outcomes, as previous studies used FML for at least 1 month pre-operatively [12,26,27].

MCP-1, also known as chemokine C-C motif ligand 2 (CCL2), is one of the key chemokines that regulate the migration and infiltration of monocytes/macrophages [28] and has a vital role in mediating leukocyte recruitment to sites of inflammation [29,30,31] in the early stages of wound healing activity. MCP-1 is expressed at higher levels in chronically inflamed eyes and is associated with increased levels of conjunctival fibroblasts and inflammatory cells [6]. It also plays a central role in coordinating the fibroproliferative stage of the wound-healing response through its angiogenic effects on the vascular endothelium and recruitment of endothelial cells to sites of vascular injury [32]. In a study by Chong et al. [8], which studied 17 different cytokines expressed in the tears of medicated glaucoma patients compared to controls who were unmedicated, MCP-1 was the only cytokine found to be significantly elevated. Furthermore, there was a significant 3-fold increase in MCP-1 concentration in the tears of eyes requiring post-operative interventions compared with those who did not require post-operative interventions following trabeculectomy. This finding linked higher MCP-1 levels with an early propensity to scar following glaucoma filtration surgery, identifying it as a possible tear biomarker to predict the potential level of post-operative scarring and thus the risk of surgical failure in patients [3,8].

In our study, we found that a 2-week course of pre-operative FML reduced MCP-1 in the tears, with the mean levels of MCP-1 reduced from 423.4 ± 523.9 pg/mL/mg at baseline to 314.1 ± 442.4 pg/mL/mg after the use of FML. This has been supported by the previous literature, where topical steroids have been found to exert beneficial effects in reducing MCP-1 levels [33] in patients with dry eyes, reversing the cellularity (i.e., macrophages, lymphocytes, mast cells) of conjunctival tissues [26] and decreasing conjunctival inflammation [27]. Interestingly, our study found a large variation in levels of MCP-1 pre-operatively, and a scatter plot showed that for those with lower MCP-1 levels (<250 pg/mL/mg) pre-operatively, their MCP-1 levels did not significantly reduce after FML administration. We postulate that this may be a reflection of a “floor effect” whereby patients with baseline lower levels of MCP-1 did not elicit any further reduction in MCP-1 levels, or it may be a reflection of FML being of weaker potency than other steroids (such as prednisolone acetate) or from a shorter duration of treatment in our study, as the potency of steroids also increases following a longer duration of treatment [34]. However, we selected FML for our study due to the better safety profile with its limited tendency to elevate IOP and reduced ocular penetrance, compared to other more potent steroids (e.g., Prednisolone acetate 1%). Furthermore, while pre-operative steroids have been used up to 4–8 weeks pre-operatively in other studies, we chose to study the outcomes of a 2-week course of FML for our study, to provide a real-world scenario in our clinical practice whereby patients may undergo surgery within a short time following listing for surgery.

Based on our posthoc observation that FML did not change the levels of MCP-1 much in patients with a baseline level of MCP-1 <250 pg/mL/mg, we performed a sub-analysis on patients with MCP-1 levels higher than 250 pg/mL/mg and found that pre-operative FML reduced the odds of requiring post-operative interventions in this group. This finding substantiates previous studies wherein the use of steroids pre-operatively improved the odds of success post-operatively [12,26,27]. Broadway et al. previously reported an improvement in surgical success in trabeculectomy surgery [26] from 50% to 81% after one month of pre-operative FML, in combination with the cessation of anti-glaucomatous medications.

Mechanisms linking reduced MCP-1 levels to improved early post-trabeculectomy outcomes may be related to a reduction in monocytes, which can lead to reduced expression of profibrotic genes and improved morphology. This is supported by a previous study [3] by Chong et al., where the application of MCP-1 receptor inhibitor (MCP-Ri) resulted in a marked reduction in the number of monocytes and monocyte-derived macrophages in a preclinical model of glaucoma filtration surgery (GFS). The expression of Collagen 1a1 and Sparc, which are heavily involved in the extracellular matrix remodeling process associated with fibrosis and scarring, was also reduced after the application of MCP-Ri. Confocal imaging of the blebs treated with MCP-Ri demonstrated preserved bleb height with characteristic subepithelial cystic spaces associated with functioning trabeculectomies in clinical studies [35,36,37], similar to changes seen following the application of mitomycin C [3]. These results suggest that reducing MCP-1 levels can result in a diminished early inflammatory response, leading to an overall reduction in postsurgical fibrosis and the maintenance of bleb survival. Further studies are warranted to confirm these observations are clinically applicable post-trabeculectomy.

The finding that pre-operative FML improves surgical success in patients with higher MCP-1 levels in our study suggests that those who may benefit most from pre-operative steroids are those with risk factors for expressing higher MCP-1 levels. As MCP-1 is produced as a response to inflammatory stimuli [38], eyes with higher MCP-1 tear levels may result from any use of glaucoma medications [8], a longer duration of glaucoma medications [8], the presence of inflamed ocular surfaces [38], moderate-to-severe dry eyes [33], or significant meibomian gland disease [39]. In our study, we also found that those who had higher MCP-1 levels in their tears pre-operatively were more likely to be older. This may reflect the observation that dry eyes and poorer ocular surfaces are more prevalent in older adults, due to risk factors such as polypharmacy, androgen deficiency, decreased blink rates, and oxidative stress in the aging eye [40]. This merits further investigations to determine the profiles of such eyes and their effects on surgical outcomes.

Our study, which is the first to be performed in a predominantly Asian population, compared to previous RCT studies, which were performed in Caucasian populations [12,41], supports the consideration of pre-operative steroids in Asian eyes undergoing trabeculectomy, since Asian eyes have also been observed to have a more aggressive post-surgical fibrotic response compared to Caucasian eyes [22,23]. Other strategies that may be used in combination with topical steroids to optimize the eye for surgery pre-operatively may include the cessation of topical anti-glaucoma medications [42], switching from preserved to non-preserved topical eyedrops [4], treating any meibomian gland disease [39], and the addition of pre-operative non-steroidal anti-inflammatory topical eyedrops [12,20].

The limitations of our study include the fact that data from the historical controls were retrospectively collected, and the group that did not receive FML pre-operatively did not have their tears collected for cytokine analysis. Therefore, it was not possible to compare the MCP-1 levels in the non-FML group with those in the FML group. The group of eyes that received pre-operative FML was relatively small in number (*n* = 36), and the sample size of the patients after propensity score matching was also small (*n* = 17). The threshold used to exclude subjects with baseline MCP-1 <250 pg/mL/mg was based on exploratory post hoc observations of our sample results, as MCP-1 levels can differ based on the specific ELISA immunoassay kit used [43], and therefore a standardized threshold limit is not available from the previous literature. The propensity matching for the FML and non-FML groups was not caliper-based, and hence this matching may present an element of bias. Nevertheless, this was the best possible matching with the available matching cohort. Moreover, the power calculation was based on a reference rate [12] and not a post-intervention rate. A larger prospective controlled study is warranted to further support these preliminary findings on the benefits of pre-operative FML to improve early post-operative outcomes in trabeculectomy.

## 5. Conclusions

In conclusion, this study showed that in our population of Asian glaucoma patients undergoing trabeculectomy and phaco-trabeculectomy, 2 weeks of therapy comprising FML eyedrops pre-operatively improved early trabeculectomy outcomes for those with higher pre-operative MCP-1 levels, with a reduction in the odds of requiring rescue interventions for impending bleb failure. Our results inform clinicians that the use of a short course of pre-operative topical steroids before trabeculectomy should be individualized and may help to improve early surgical outcomes.

## Figures and Tables

**Table 1:** Study population demographics.

	FML Group (*n* = 36)	Non-FML Group (*n* = 307)	*p*-Value
Mean (SD) age, years	65.6 (11.3)	67.9 (12.1)	0.278
Gender, male, *n* (%)	19 (52.8)	200 (65.1)	0.143
Race			
Chinese	29 (69.4%)	257 (83.7)	0.035
Malay	6 (16.7%)	15 (4.9)	
Indian	5 (13.9%)	25 (8.1)	
Others	0 (0)	10 (3.3)	
Type of medication			
PGA	33 (91.7)	299 (97.4)	0.663
Timolol	32 (88.9)	268 (87.3)	0.784
Brimonidine	22 (61.1)	224 (73.0)	0.134
CAI	25 (65.8)	218 (71.0)	0.518
Duration of medication prior to surgery (months)	*n* = 35		
Mean (SD)	32.9 (30.2)	67.2 (57.8)	<0.001
Range	1–123	0–208	
Number of different medications at time of surgery			
0	0	2 (0.7)	0.142
1	1 (2.8)	11 (3.6)	
2	6 (16.7)	44 (14.3)	
3	18 (47.2)	90 (29.3)	
4	12 (33.3)	160 (52.1)	
Glaucoma diagnosis			
POAG	8 (22.2)	170 (55.4)	<0.001
NTG	20 (55.6)	50 (16.3)	
PACG	7 (19.4)	85 (27.7)	
Others	1 (2.8) (JOAG)	2 (0.7) (2 eyes with primary congenital glaucoma)	
Surgery type, *n* (%)			
Trabeculectomy	12 (33.3)	85 (27.7)	0.477
Phaco-trabeculectomy	24 (66.7)	222 (72.3)	
Intra-operative complications	1 (2.8)	2 (0.7)	0.213

Data presented as number (%) or mean (standard deviation [SD]), as appropriate.

**Table 2:** (**A**) Outcome measures determined at 6 months after trabeculectomy of patients with and without FML (all patients). (**B**) Propensity score-matched univariate analysis matching for age, gender, ethnicity, diagnosis, duration of glaucoma eyedrops, and type of surgery (excluding those with pre-operative MCP-1 levels less than 250 pg/mL/mg).

(A)				
	FML Group (*n* = 36)	Non-FML Group (*n* = 307)		*p*-Value
Any IOP-lowering medication	7 (19.4)	87 (28.3)		0.258
Any needling performed	10 (27.8)	86 (26.7)		0.888
Number of needlings performed (RANGE)	1.2 (0.63) (1–3)	1.5 (0.74) (1–4)		0.020
Any further surgical intervention	1 (2.8)	2 (0.7)		0.213
Any intervention (needling, surgery, and/or medication)	13 (36.1)	140 (45.6)		0.279
Final IOP (RANGE)	11.7 (3.9) (1–24)	11.8 (4.2) (4–32)		0.892
**(B)**				
**FML (*n* = 34)**	**Odds Ratio**	**CI Lower**	**CI Upper**	***p*-Value**
Any IOP-lowering medication *n* = 17 (1:1)	0.69	0.13	3.72	0.672
Any needling performed *n* = 17 (1:1)	0.19	0.03	1.11	0.065
Number of needlings performed	0.23	0.05	1.04	0.057
Any intervention (needling, surgery, and/or medication) *n* = 17 (1:1)	0.22	0.05	0.946	0.042
Final IOP *n* = 17 (1:1)	1.07	0.91	1.72	0.429

Data presented as number (%) or mean (standard deviation [SD]), as appropriate.

**Table 3:** Characteristics of patients with higher MCP levels (250 pg/mL/mg or more) vs. those with lower MCP levels (<250 pg/mL/mg).

	Higher MCP Levels with Pre-treatment (*n* = 17)	Lower MCP Levels (*n* = 19)	*p*-Value
Mean (SD) age, years	71.88 (6.5)	59.95 (11.8)	**<0.001**
Gender, male *n* (%)	10 (58.8)	9 (47.4)	0.492
Race			
Chinese	11 (64.7)	14 (73.7)	0.353
Malay	2 (11.8)	4 (21.1)	
Indian	4 (23.5)	1 (5.3)	
Duration of medication prior to surgery (months)			
Mean (SD)	31.3 (31.2)	34.4 (31.0)	
(Range)	(1–104)	(1–123)	0.385
Number of different medications at time of surgery			
0	14 (82.4)	15 (78.9)	
1	1 (5.9)	0	0.408
2	0	0	
3	2 (11.8)	1 (5.3)	
4	0	3 (15.8)	
Glaucoma diagnosis			
POAG (1)	5 (29.4)	3 (15.8)	
NTG (2)	8 (47.1)	12 (63.2)	0.585
PACG (3)	4 (23.5)	3 (15.8)	
Others (4)	0	1 (5.3)	
**Post-Operative Outcomes**			
Any IOP-lowering medication			
Yes	3 (17.6)	4 (21.1)	1.0
No	14 (82.4)	15 (78.9)	
Any needling performed			
Yes	2 (11.8)	8 (42.1)	0.065
No	15 (88.2)	11 (57.2)	
Number of needlings performed	1 (0.0) Range (1–1)	1.25 (0.7) Range (1–3)	0.640
Any further surgical intervention			
Yes	0	1 (5.3)	
No	17 (100)	18 (94.7)	1.0
Any intervention (needling, surgery, or medication)			
Yes	4 (23.5)	9 (47.4)	
No	13 (76.5)	10 (52.6)	0.137
Final IOP	11.88 (3.3) Range (7–18)	11.58 (4.4) Range (1–24)	0.821

Data presented as number (%) or mean (standard deviation [SD]), as appropriate.

## Data Availability

The datasets generated and/or analyzed during the current study are not publicly available as they contain patient data, but they are available from the corresponding author on reasonable request.

## Abbreviations

The following abbreviations are used in this manuscript:

FML	Fluorometholone
CCL-2	Chemokine C-C motif ligand 2
MCP-1	Monocyte Chemoattractant Protein 1
RCT	Randomized Controlled Trial
IOP	Intraocular pressure
